# Identification of a competing endogenous RNA network associated with prognosis of pancreatic adenocarcinoma

**DOI:** 10.1186/s12935-020-01243-6

**Published:** 2020-06-11

**Authors:** Wanqing Weng, Zhongjing Zhang, Weiguo Huang, Xiangxiang Xu, Boda Wu, Tingbo Ye, Yunfeng Shan, Keqing Shi, Zhuo Lin

**Affiliations:** 1 Zhejiang Provincial Key Laboratory, The First Affiliated Hospital of Wenzhou Medical University, Wenzhou, 325000 Zhejiang People’s Republic of China; 2 Precision Medicine Center Laboratory, The First Affiliated Hospital of Wenzhou Medical University, Wenzhou, 325000 Zhejiang People’s Republic of China; 3 Department of Hepatobiliary Surgery, The First Affiliated Hospital of Wenzhou Medical University, Wenzhou, 325000 Zhejiang People’s Republic of China; 4 Department of Liver Diseases, The First Affiliated Hospital of Wenzhou Medical University, Wenzhou, 325000 Zhejiang People’s Republic of China

**Keywords:** Pancreatic adenocarcinoma, Competing endogenous RNA network, Time-dependent receiver operating characteristic

## Abstract

**Background:**

Emerging evidence suggests that competing endogenous RNAs plays a crucial role in the development and progress of pancreatic adenocarcinoma (PAAD). The objective was to identify a new lncRNA-miRNA-mRNA network as prognostic markers, and develop and validate a multi-mRNAs-based classifier for predicting overall survival (OS) in PAAD.

**Methods:**

Data on pancreatic RNA expression and clinical information of 445 PAAD patients and 328 normal subjects were downloaded from The Cancer Genome Atlas (TCGA), International Cancer Genome Consortium (ICGC) and Genotype-Tissue Expression (GTEx). The weighted correlation network analysis (WGCNA) was used to analyze long non-coding RNA (lncRNA) and mRNA, clustering genes with similar expression patterns. MiRcode was used to predict the sponge microRNAs (miRNAs) corresponding to lncRNAs. The downstream targeted mRNAs of miRNAs were identified by starBase, miRDB, miRTarBase and Targetscan. A multi-mRNAs-based classifier was develop using least absolute shrinkage and selection operator method (LASSO) COX regression model, which was tested in an independent validation cohort.

**Results:**

A lncRNA-miRNA-mRNA co-expression network which consisted of 60 lncRNAs, 3 miRNAs and 3 mRNAs associated with the prognosis of patients with PAAD was established. In addition, we constructed a 14-mRNAs-based classifier based on a training cohort composed of 178 PAAD patients, of which the area under receiver operating characteristic (AUC) in predicting 1-year, 3-year, and 5-year OS was 0.719, 0.806 and 0.794, respectively. The classifier also shown good prediction function in independent verification cohorts, with the AUC of 0.604, 0.639 and 0.607, respectively.

**Conclusions:**

A novel competitive endogenous RNA (ceRNA) network associated with progression of PAAD could be used as a reference for future molecular biology research.

## Introduction

Pancreatic adenocarcinoma (PAAD), a common digestive tract tumor, is the eighth leading cause of cancer death in the world. The 5-year survival rate for pancreatic cancer is only 5%, due to its rapid metastasis, high degree of malignancy and difficulty in early diagnosis [1]. Many studies have shown that chronic pancreatitis, gallstones, alcohol, and smoking were the most common risk factors for PAAD [2–5]. Serum carbohydrate antigen (CA 19-9) is the most common tumor marker of PAAD. But due to its low sensitivity and accuracy in clinical practice, many patients cannot be diagnosed in the early stage of cancers [6, 7]. Although much progress has been made in early diagnosis, including new diagnostic markers, such as: sTAR, miR-25, etc., but the overall survival rate for PAAD has not improved in recent years [8, 9]. Therefore, it is urgent to find more biomarkers as early diagnostic indicators for PAAD, guiding the treatment of the disease.

During the occurrence and development of diseases, multiple interacting RNAs could be changed. So figuring out the mechanism of RNA interactions would help us to understand pathogenesis of diseases [10]. In recent years, many papers have studied and reported the interaction between non-coding RNA and mRNA based on the ceRNA hypothesis. In general, a variety of RNAs are involved in ceRNA regulatory networks, including lncRNA, miRNA, circular RNA and mRNA. MiRNA can bind to the 3′ untranslated region of mRNA in order to inhibit the translation of mRNA [11]. The ceRNA hypothesis suggests that lncRNA competitively binds with microRNAs through microRNA response elements (MREs) and inhibits the down-regulation of mRNA translation by miRNAs [12]. Moreover, the role of lncRNA-miRNA-mRNA ceRNA regulatory networks has been revealed in different diseases, such as chronic myeloid leukemia, cardiovascular diseases, diabetic cataract, and myocardial infarction [13–16], which might impact the prognosis of the diseases. However, on the basis of high-throughput sequencing and large samples, only a few literatures have reported the role of ceRNA in PAAD.

**Fig. 1:**
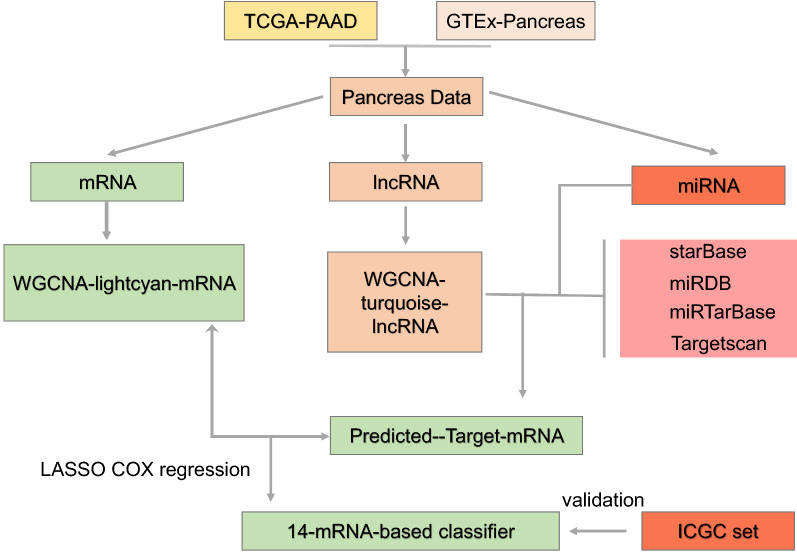
The workflow of this work

In the current study, therefore, we aimed to identify a new lncRNA-miRNA-mRNA network as prognostic markers for PAAD. The RNA-Seq data of 178 and 267 cases of PAAD tissues were obtained from TCGA and ICGC, and 328 cases of normal pancreas tissues from GTEx, respectively. Then, mRNAs and lncRNAs between the normal samples and PAAD patients were applied to WGCNA to enrich modules which were most related to PAAD. The target genes of miRNAs were predicted by the staBase, miRDB, miRTarBase and Targetscan. A multi-mRNAs-based classifier and a lncRNA-miRNA-mRNA ceRNA network containing 60 lncRNAs, 3 miRNAs and 3 mRNAs influencing the prognosis of PAAD were developed (Fig. 1).

## Results

### Different genes expression between the normal samples and PAAD tissues

**Fig. 2:**
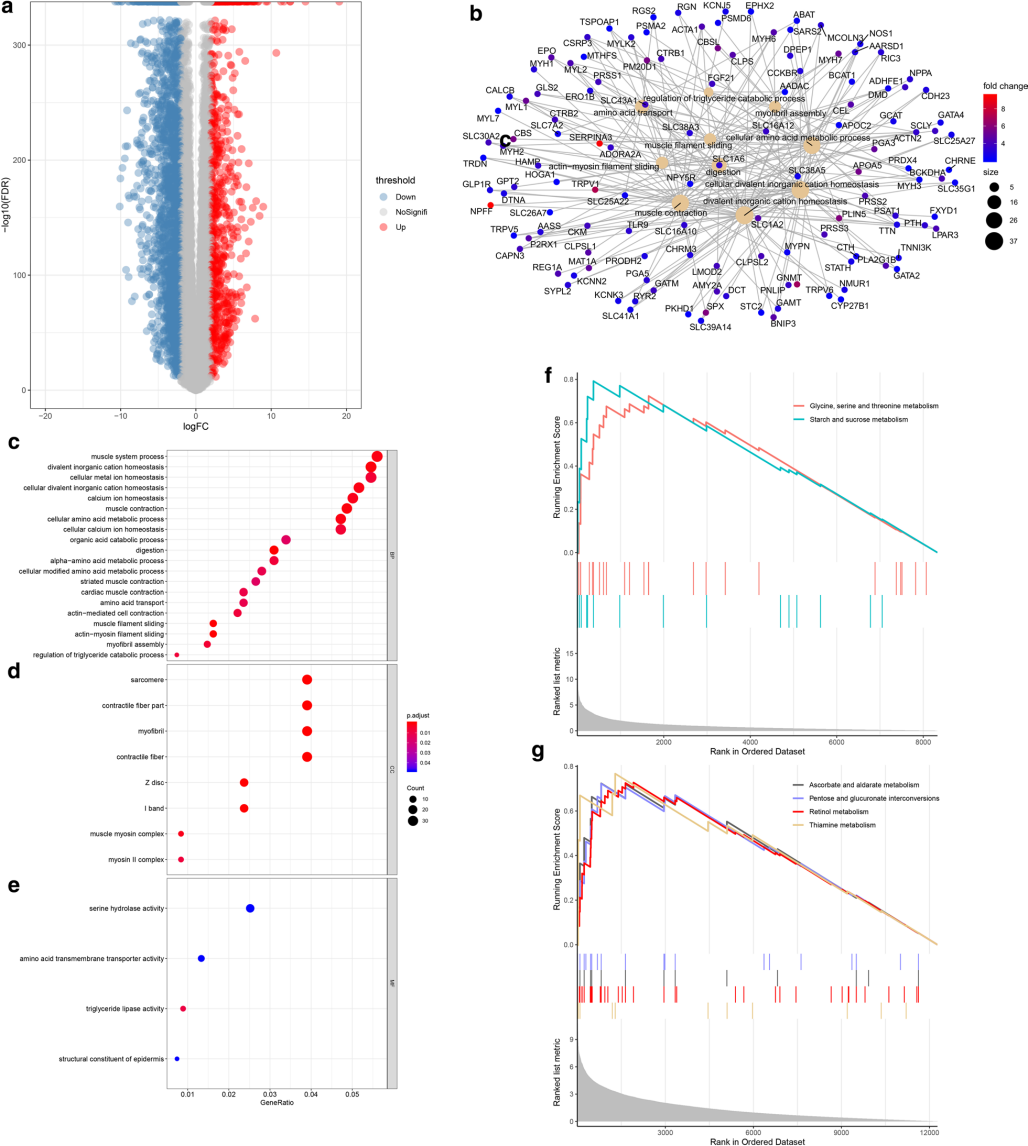
Different gene expression between the normal samples and PAAD tissues is analyzed. **a** The volcano map showing the differentially expressed mRNAs from TCGA and GTEx. Red spots represent up-regulated genes, and blue spots represent down-regulated genes. **b** Interaction network of the significantly up-regulated mRNAs in BP. **c**–**e** Plot of up-regulated mRNA enrichment in BP (**c**), CC (**d**) and MF (**e**) in GO analysis. **f**, **g** KEGG-GSEA was applied for signal pathway analysis

We collected RNA-seq data of pancreatic tissue from 178 PAAD patients and 328 normal samples from TCGA and GTEx. EdgeR was used to normalized the gene read counts to the trimmed mean of M values (TMM). As shown in volcano map, with − log10 (false discovery rate, FDR) as the y-coordinate and log2 (fold change, FC) as the x-coordinate, 822 mRNAs were up regulated and 2362 mRNAs were down regulated (Fig. 2a). We applied these up-regulated mRNAs with Gene Ontology (GO) to investigate their potential function. In biological processes (BP), these up-regulated mRNAs were enriched in the muscle system process, divalent inorganic cation homeostasis, cellular metal ion homeostasis, cellular divalent inorganic cation homeostasis (Fig. 2c). As shown in Fig. 2d, e, the cellular component (CC) and molecular function (MF) analysis results also showed specifically up-regulated mRNAs enrichment. Gene symbols of up-regulated mRNAs and their interaction network in BP were shown in Fig. 2b. To investigate the role of these genes in various biological pathways, they were analyzed using Kyoto Encyclopedia of Genes and Genomes (KEGG)-Gene Set Enrichment Analysis (GSEA). The results showed that up-regulated genes were enriched in glycine, serine and threonine metabolism and starch and sucrose metabolism, while down-regulated genes were enriched in ascorbate and aldarate metabolism, pentose and glucuronate interconversions, retinol metabolism and thiamine metabolism (Fig. 2f, g).

### MRNAs modules

**Fig. 3:**
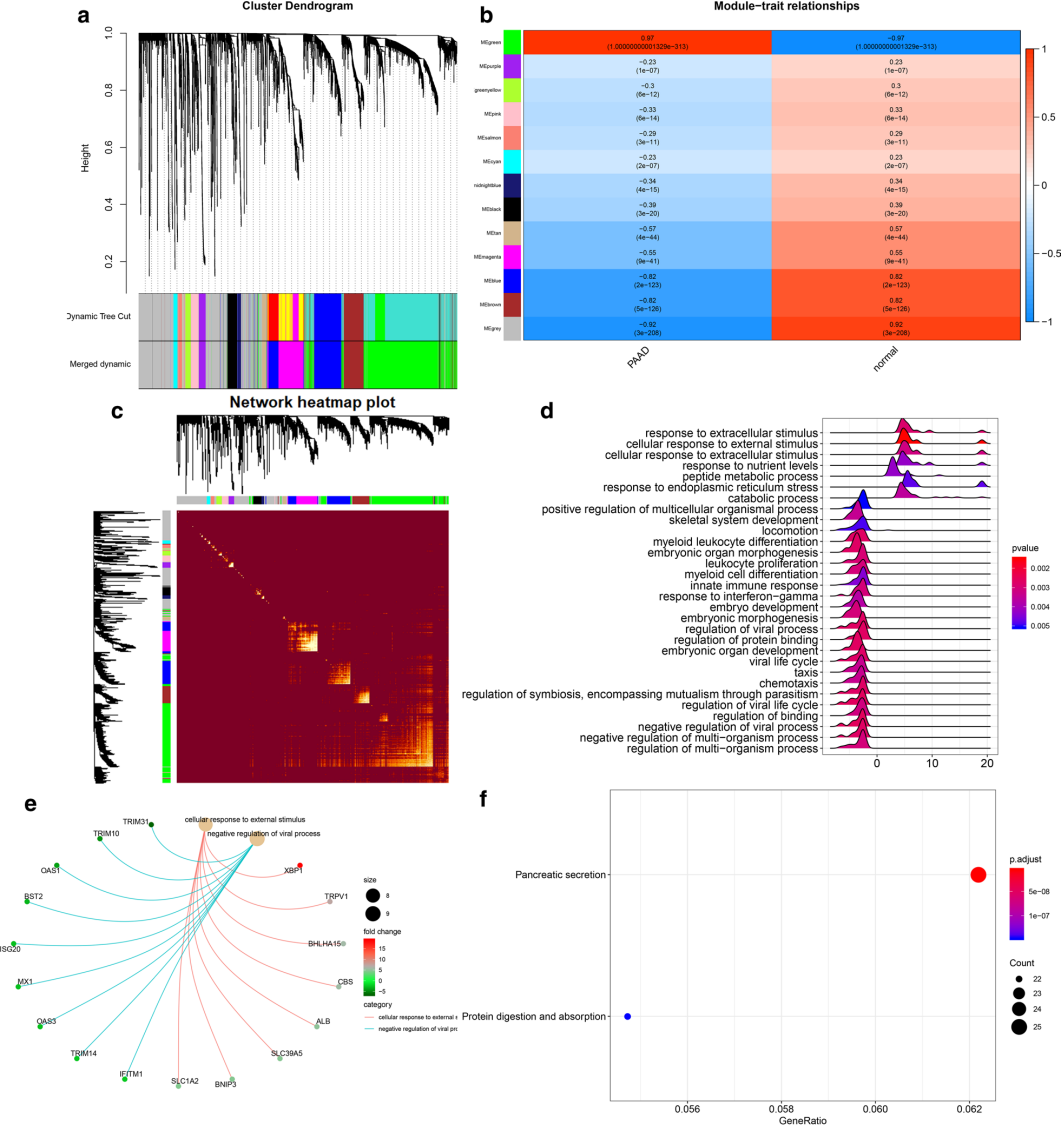
The mRNAs modules were analyzed by WGCNA. **a** Cluster dendrogram of the co-expression network modules was produced based on topological overlap in the mRNAs. **b** The relation of genes in modules between PAAD tissues and normal samples was investigated. **c** Network heatmap plot of topological overlap in the gene network. **d**, **e** GO-GSEA displayed the gene symbols and gene interaction in green module. **f** KEGG analysis was used to investigate the pathway enrichment in green module

As shown in Fig. 3a, we used WGCNA and set the thresholds to soft power 12–30 and module size cut-25. Therefore, we divided the 3184 differentially expressed mRNAs into different modules according to the expression pattern, and a total of 13 co-expressed gene modules were obtained (Fig. 3b). Figure 3c showed the corresponding heatmap plot of topological overlap matrix (TOM). Among the 13 gene co-expression modules, green module was the most relevant to the clinical characteristics of PAAD, containing 981 genes (Fig. 3c). Then we performed GO-GSEA analysis of the genes in this gene module (Fig. 3d, e), indicating that the genes were significantly related to extracellular stimulus, nutrient levels and peptide metabolic process. As shown in Fig. 3f, KEGG analysis showed that genes of green module were enriched in pancreatic secretion.

### LnRNAs modules

**Fig. 4:**
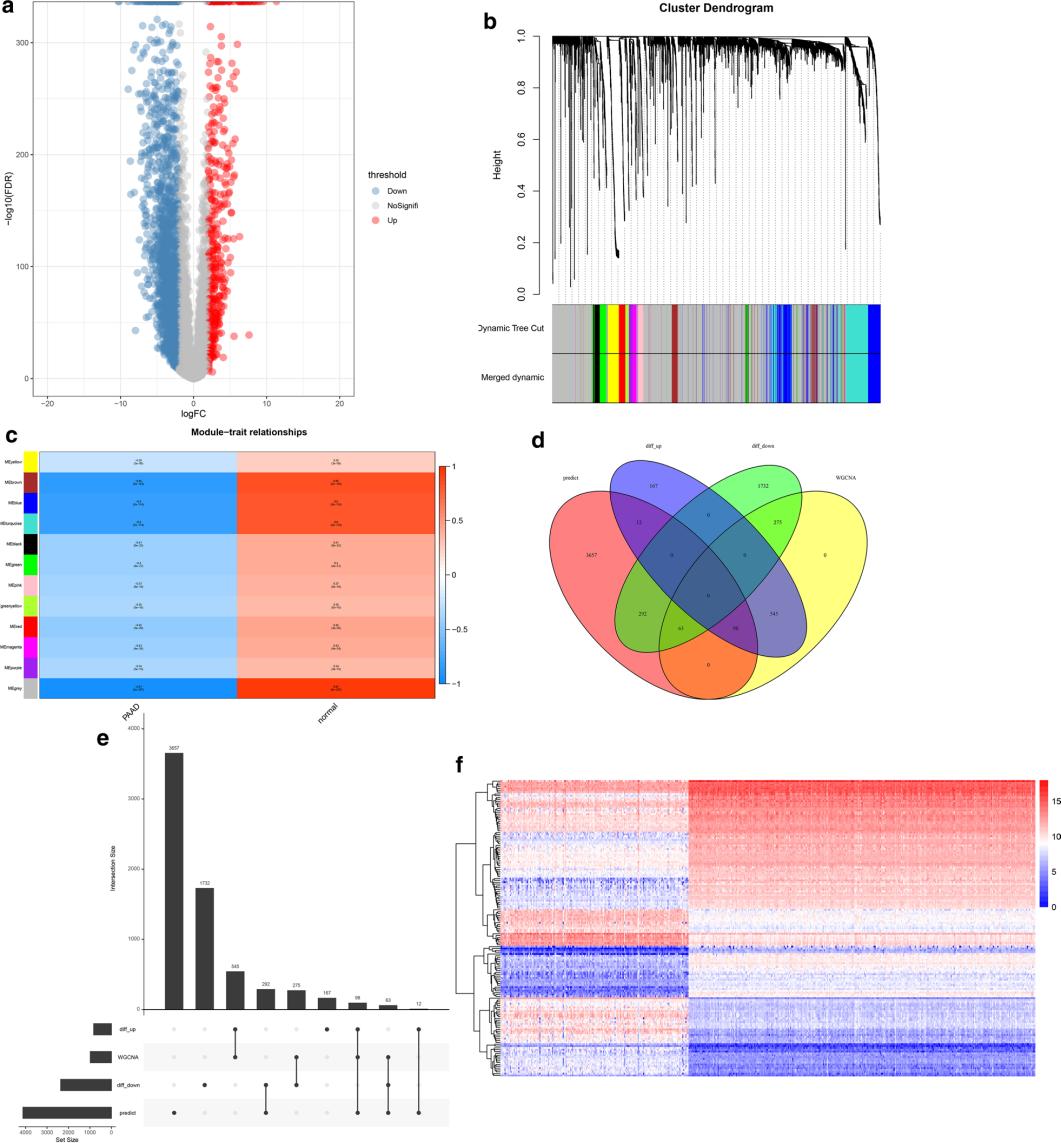
LnRNAs modules were analyzed by WGCNA. **a** The volcano map showing the differentially expressed lncRNAs from TCGA and GTEx. Red spots represent up-regulated genes, and blue spots represent down-regulated genes. **b** Cluster dendrogram of the co-expression network modules was produced based on topological overlap in the lncRNAs. **c** The relation of lncRNAs in modules between PAAD tissues and normal samples was investigated. **d**, **e** The overlapped target mRNAs obtained by overlapping the significantly up-regulated mRNAs, significantly down-regulated mRNAs, WGCNA-green module and the predicted target mRNAs were analyzed. **f** The heatmap showing the expression of 161 mRNAs in 178 PAAD patients and 328 normal samples

The volcano map of lncRNA showed that 427 lncRNA were up-regulated and 2261lncRNA were down-regulated (Fig. 4a). WGCNA was performed to analyze the co-expression of the up-regulated lncRNA, and a total of 12 modules were obtained (Fig. 4b). Among them, grey module which contains 423 genes, has the strongest correlation with PAAD patients (Fig. 4c). Then we used miRcode to predict the sponge miRNAs corresponding to the 423 genes, and overlapped with the 400 highest expressed miRNAs in the patient miRNA-seq downloaded from TCGA. There were overlapped 325 miRNAs. Through starBase, miRDB, miRTarBase and Targetscan, 4122 downstream targeted mRNAs of the 325 overlapping miRNAs were obtained. Taken together, we obtained four sets of mRNAs, including: 822 significantly up-regulated mRNAs and 2362 significantly down-regulated mRNAs by edgeR analysis, 981 mRNAs in the WGCNA-green module, and 4122 predicted target mRNAs. After overlapping the four groups of mRNAs, we got 98 up-regulated mRNAs and 63 down-regulated mRNAs (Fig. 4d, e). Expressions of 161 mRNAs in 178 PAAD patients and 328 normal samples were displayed in heatmap (Fig. 4f).

### Multi-mRNAs-based classifier

**Fig. 5:**
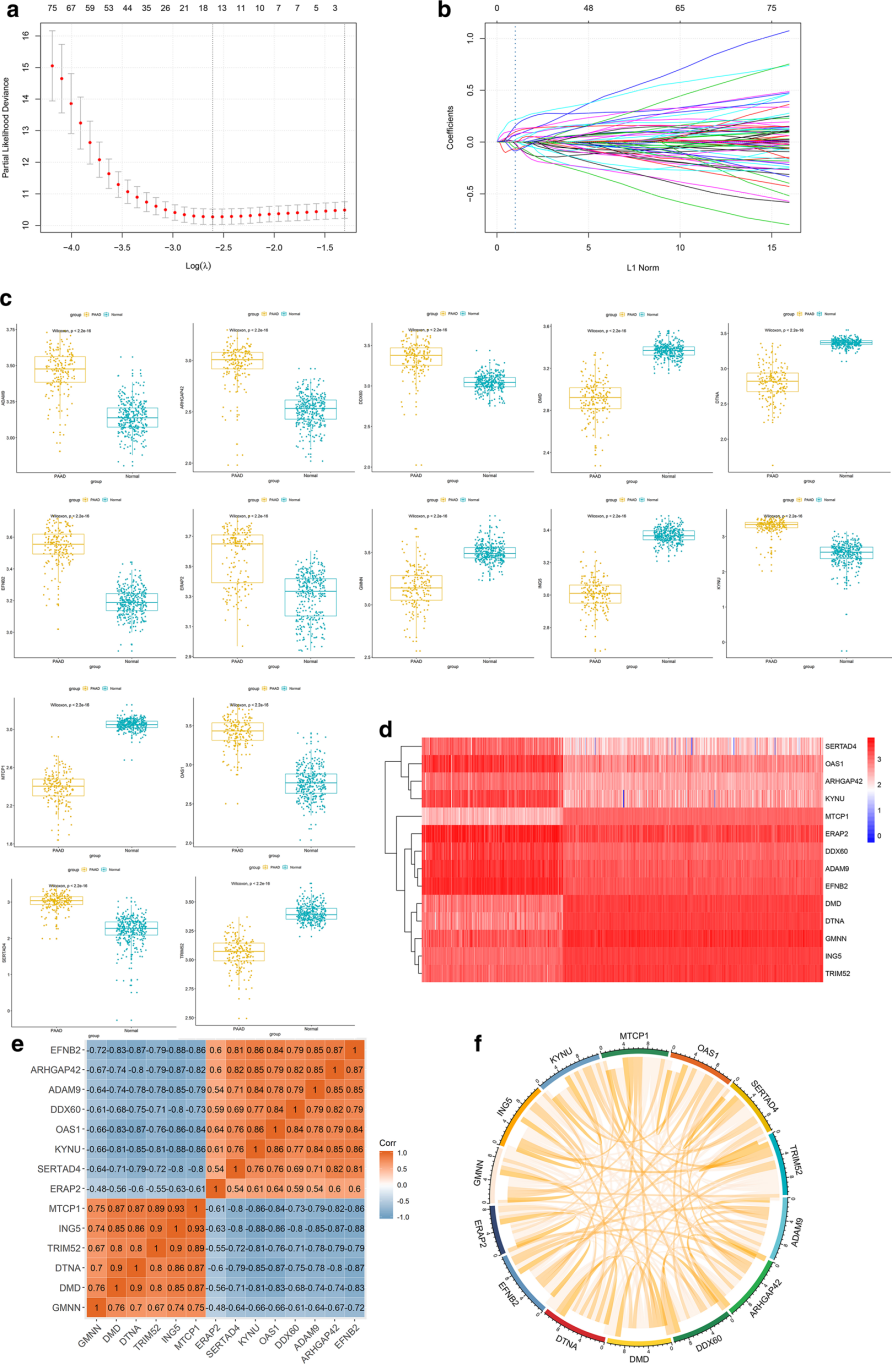
Genetic screening for multi-mRNAs-based classifier. **a** LASSO coefficient profiles of the 161 mRNAs. A vertical line is drawn at the value chosen by 13-fold cross-validation. **b** Ten-time cross-validation for tuning parameter selection in the LASSO model. **c** The expression of 14 selected genes between PAAD patient samples and normal samples was shown. **d** The expression heatmap of the 14 genes in high risk or low risk group. **e**, **f** The expression relationship of the 14 genes

**Fig. 6:**
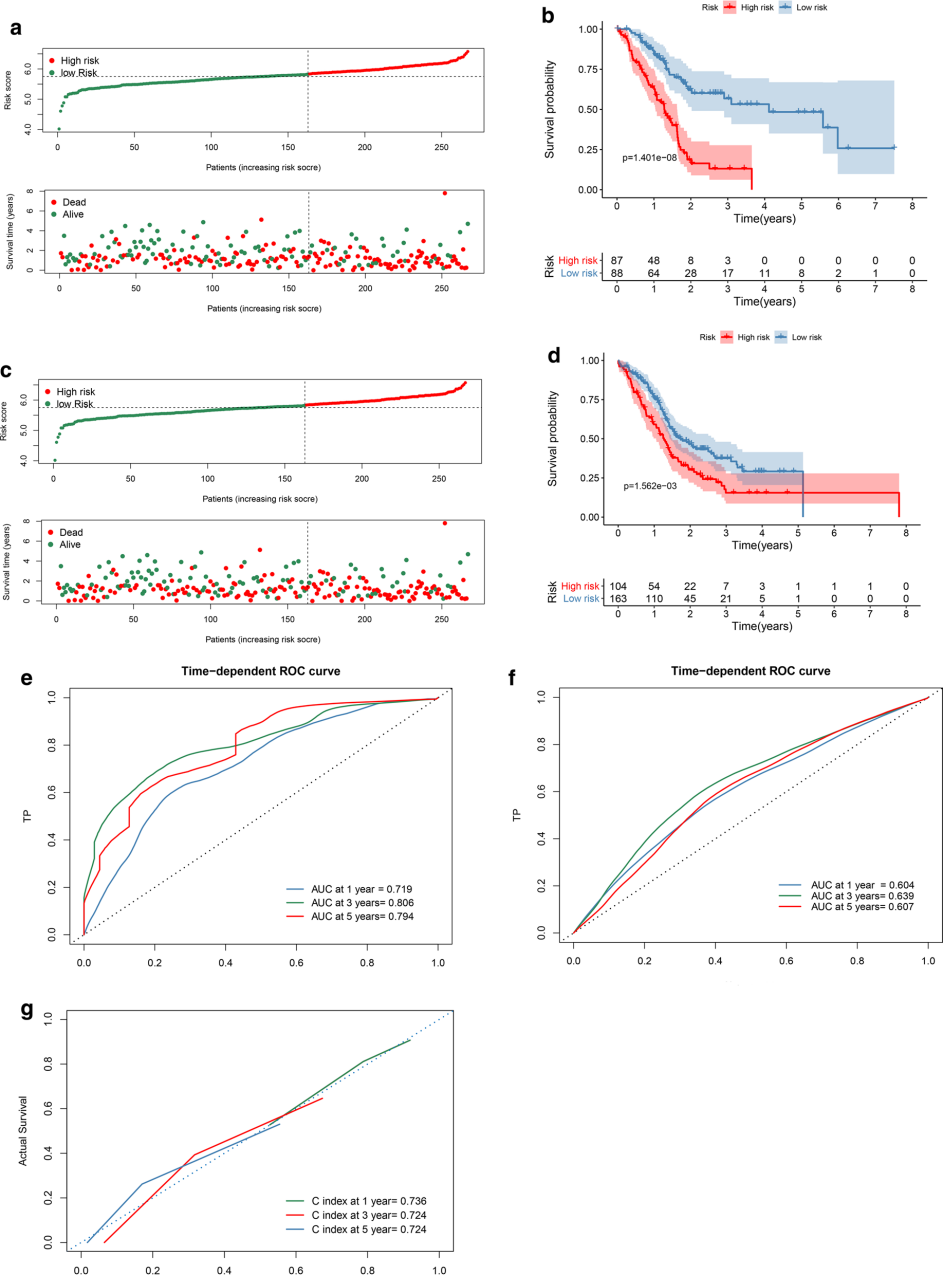
Multi-mRNAs-based classifier. **a**, **c** PAAD patients in the training cohort and the validation cohort were classified into predicted low and high-risk groups according to the multi-mRNAs-based classifier. **b**, **d** Kaplan–Meier survival analysis of the multi-mRNAs-based classifier was performed. **e**, **g** Time-dependent ROC curve and calibration curves of the multi-mRNAs-based classifier in the training cohort. **f** Time-dependent ROC curve of the multi-mRNAs-based classifier in the validation cohort

In order to construct a multi-mRNAs-based classifier for predicting OS in PAAD, 178 PAAD patients from TCGA were included in the training cohort, and 267 PAAD patients from ICGC were included in the validation cohort. LASSO COX regression method was used to identifying the mRNAs for predicting OS in the training cohort, and finally 14 mRNAs were selected (Fig. 5a, b). As shown in Fig. 5c, ADAM9, ARHGAP42, DDX60, EFNB2, ERAP2, GMNN, KYNU, OAS1 and SERTAD4 were up-regulated while DMD, DTNA, ING5, MTCP1 and TRIM52 were down-regulated in PAAD (Fig. 5d). The correlation between these 14 genes was shown in Fig. 5e, f. We established a classifier for the OS of PAAD patients based on 14 mRNAs = 0.006963639 * EXP(ADAM9) + 0.069425086 * EXP(ARHGAP42) + 0.077659645 * EXP(DDX60) − 0.029916764 * EXP(DMD) − 0.052925385 * EXP(DTNA) + 0.21956021 * EXP(EFNB2) + 0.101980826 * EXP(ERAP2) + 0.165217493 * EXP(GMNN) − 0.068491015 * EXP(ING5) + 0.122589990 * EXP(KYNU) − 0.017670448 * EXP(MTCP1) + 0.008851918 * EXP(OAS1) + 0.028523489 * EXP(SERTAD4) − 0.08385264 * EXP(TRIM52). EXP (mRNA) = Log2 (expression value of mRNA). All PAAD patients in the training cohort and validation cohort were stratified into high and low risk groups according to the 14-mRANs-based classifier (Fig. 6a, c). In addition, we used KEGG-GSEA to analyze gene enrichment in the high-risk group of the training cohort, the results show that the main enrichment of genes associated with tumor pathway, such as adheres junction, P53 signaling pathway, pancreatic cancer, TGF-β signaling pathway and so on (Additional file 1: Figure S1).

According to the survival curve, patients in the low-risk group have a more satisfactory OS than the high-risk group (P < 0.0001, Fig. 6b, d). In the training cohort, there was no significant difference in the distribution of clinical data between the low-risk group and the high-risk group (Table 1). Time dependent receiver operating characteristic (tdROC) was used to analyze the accuracy of the classifier in predicting 1-year, 3-year, and 5-year OS in training cohort and validation cohort. The AUC in the training volume was 0.719, 0.806, and 0.794, and the AUC in the validatuin cohort was 0.604, 0.639, and 0.607 (Fig. 6e, f). The average predicted probability (predicted survival rate) and Kaplan–Meier estimated (observed survival rate) were plotted to evaluate the accuracy of the prediction model, where the dotted line represented the ideal reference line corresponding to the predicted survival rate and actual survival rate. The calibration curves of 1-year, 3-year and 5-year survival probability based on 14-mRNAs-classifier were in good agreement with the actual observed values. The C-index of 1-year, 3-year, and 5-year were 0.736, 0.724 and 0.724 respectively, indicating that the prediction model has high accuracy (Fig. 6g). Univariate cox analysis also showed that Grade (HR: 1.537, 95% CI 0.994–2.378; P = 0.053), T-classification (HR: 2.194, 95% CI 1.131–4.254; P = 0.020) and multi-mRNAs-based classifier (HR: 10.119, 95% CI 5.134–19.948; P < 0.0001) were powerful and independent influencing factors for OS (Table 2).

**Table 1:** Correlations between risk score of the 14-mRNAs-based classifier with overall survival and clinicopathological characteristics in training cohort

Clinicopathological variables	Number of patients	High risk	Low risk	P value
Training cohort				
Age				
≤ 65	81 (46.3%)	39 (48.1%)	42 (51.9%)	
> 65	94 (53.7%)	48 (51.1%)	46 (48.9%)	0.762
M				
M0	78 (95.1%)	45 (57.7%)	33 (42.3%)	
M1	4 (4.9%)	2 (50%)	2 (50%)	1.0
N				
N0	49 (28.8%)	20 (40.8%)	29 (59.2%)	
N1	121 (71.2%)	67 (55.4%)	54 (44.6%)	0.093
Stage				
I + II	165 (95.4%)	82 (49.7%)	83 (50.3%)	
III + IV	8 (4.6%)	13 (62.5%)	10 (37.5%)	0.720
T				
T1 + T2	30 (17.3%)	12 (40.0%)	18 (60.0%)	
T3 + T4	143 (82.7%)	75 (52.4%)	68 (47.6%)	0.234
Grade				
G1&2	123 (71.1%)	55 (44.7%)	68 (55.3%)	
G3&4	50 (28.9%)	31 (62.0%)	19 (38.0%)	0.045
Gender				
Male	95 (54.3%)	42 (44.2%)	53 (55.8%)	
Female	80 (45.7%)	45 (56.3%)	35 (43.8%)	0.130

**Table 2:** Univariate COX analyses of the mRNAs-based classifier for overall survival

Prognostic parameter	Univariate analysis		
	HR	95% CI	P value
Training cohort			
Age (> 65 vs. ≤ 65)	1.403	0.922–2.135	0.114
Gender (male vs. female)	1.234	0.817–1.862	0.318
Grade (G3&4 vs. G1&2)	1.537	0.994–2.378	0.053
Tumor stage (III + IV vs. I + II)	0.734	0.231–2.329	0.600
T classification (T3 + T4 vs. T1 + T2)	2.194	1.131–4.254	0.020
Multi-mRNAs-based classifier (high vs. low risk)	10.119	5.134–19.948	< 0.0001

*HR* hazard ratio, *CI* confidence interval

### LncRNA-miRNA-mRNA ceRNA network

**Fig. 7:**
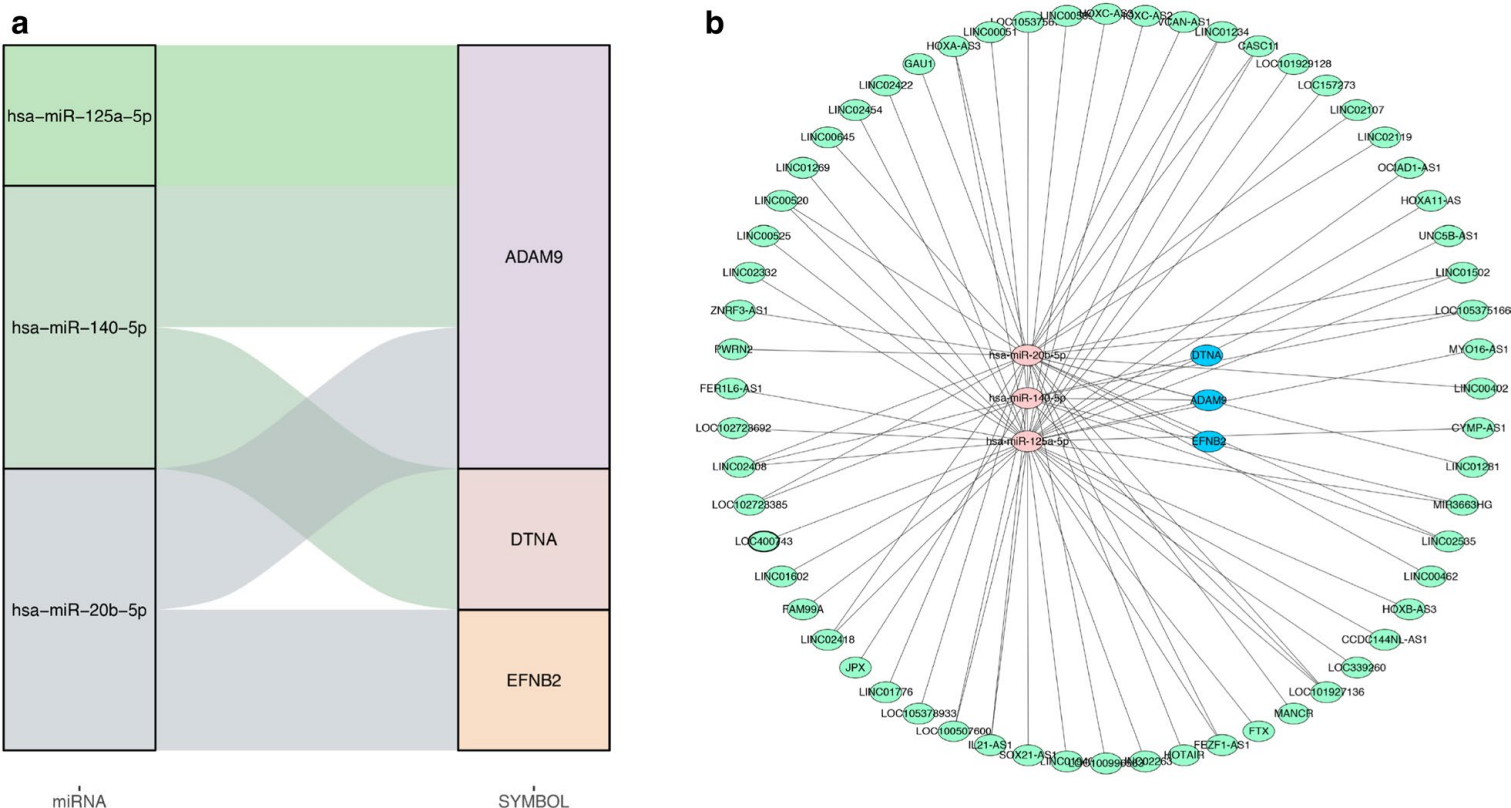
LncRNA-miRNA-mRNA ceRNA network. **a** The interaction network of 10 target genes and their corresponding miRNA. **b** The lncRNA-miRNA-mRNA ceRNA network. The green hexagon represents lncRNA, the red diamond represents mirRNA, and the blue oval represents mRNA

In order to build a gene co-expression network, we matched 14 mRNAs with their upstream miRNAs, and finally found the significant correlation between hsa-mir-125a-5p, has-mir-140-5p, hsa-mir-20b-5p and downstream mRNAs (ADAM9, DTNA and EFNB2). Hsa-mir-125a-5p targets ADAM9, has-mir-140-5p targets ADAM9 and DTNA, and hsa-mir-20b-5p targets ADAM9 and EFNB2 (Fig. 7a). As mentioned above, 427 up-regulated lncRNAs and 2261 down-regulated lncRNAs were identified. The 2688 lncRNAs were overlapped with the predicted lncRNAs of 3 miRNAs, and a total of 60 lncRNAs were obtained. Finally, the lncRNA-miRNA-mRNA co-expression network of PAAD was composed of 60 lncRNAs, 3 miRNAs and 3 mRNAs (Fig. 7b).

### Multi-lncRNAs-mRNAs-based classifier

To assess whether the classifier composed of multiple type RNA has better prediction capability, we tried to use lncRNA and mRNA to establish a multi-RNA type classifier. The LASSO COX regression method was applied to 60 lncRNAs in the ceRNA network and 14 mRNAs in the mRNAs-based classifier, and finally screened out 16 RNAs. Then a 16-genes-based classifier was constructed (Additional file 2: Figure S2A, B). The 16-genes-based classifier = 0.097341752 * EXP(CASC11) + 0.034862251 * EXP(LINC00462) + 0.038317729 * EXP(LINC00520) + 0.008667157 * EXP(LINC01269) + 0.118122095 * EXP(LINC01776) + 0.055449539 * EXP(LINC01940) + 0.005476430 * EXP(MANCR) + 0.015533127 * EXP(UNC5B.AS1) + 0.086004600 * EXP(ARHGAP42) + 0.088792744 * EXP(DDX60) + 0.077569015 * EXP(EFNB2) + 0.066588930 * EXP(ERAP2) + 0.201239919 * EXP(GMNN)-0.051922288 * EXP(ING5) + 0.042613806 * EXP(KYNU) + 0.038130832 * EXP(SERTAD4). The classifier was applied to 178 PAAD patients in the TCGA database and divided into a high-risk group and a low risk group according to the threshold value of the median score. The survival curves of the high-risk and low-risk groups were significantly different (Additional file 2: Figure S2C). In the time-dependent ROC curve, the classifier could effectively predict the 1-year, 3-year and 5-year survival rates of patients, with the AUC of 0.778, 0.857 and 0.819, respectively (Additional file 2: Figure S2D). Calculated by the TIMEROC software package and the survival software package, the differences in AUC of 1 year, 3 years, and 5 years of the two classifiers were not statistically significant, and both the P-value and adjusted P-value were greater than 0.05 (Table 3).

**Table 3:** Comparison of AUC of 14-mRNAs-based classifier and 16-genes-based classifier

Group	AUC at 1 year	AUC at 3 years	AUC at 5 years
P value	0.06958159	0.2951822	0.5310802
Adjusted P value	0.19008718	0.6394491	0.8917302

## Methods

### Patients data

We first downloaded RNA-seq, miRNA-seq and clinical information data of pancreatic tissue of PAAD patients from TCGA data repository (up to September 6, 2019, https://portal.gdc.cancer.gov/), which are publicly available resources. After excluding samples without sufficient data, we obtained a total of 178 tumors samples as a training cohort. We also obtained RNA-seq data of 267 PAAD patients from ICGC data base (up to September 6, 2019, https://icgc.org/) as a validation cohort. RNA-seq data of 328 normal subjects were downloaded from GTEx database (up to September 6, 2019, https://www.gtexportal.org/home/index.html). The genes were re-annotated by the rtracklayer package of The R software (Version3.6.1). The gene annotation file “Homo_sapiens. GRCh38.91.CRH.gtf” was downloaded from the Ensembl Genomes website (http://asia.ensembl.org/index.html).

### Identification of differentially expressed genes

To distinguish differentially expressed genes in mRNA and lncRNA data sets, the edgR package was performed through R software version 3.6.1. All q values use FDR to correct the statistical significance of the multiple test. In the result, if the gene of|log2FC| > 2 and FDR < 0.05, the gene will be retained for the following analysis. The ggplot2 package in R platform was used to generate the volcano plot, so as to more intuitively show the difference in mRNA and lncRNA expressions.

### Functional enrichment analysis

We used GO to analyze the enrichment of differentially expressed mRNA from biological process (BP), cellular component (CC) and molecular function (MF) [17]. KEGG-GSEA was used to look for gene enrichment in metabolic pathways, and the significance level was P < 0.05 [18].

### Weighted gene co-expression network analysis (WGCNA)

WGCNA, a system biology method, can identify highly synergistic gene sets and can analyze the genes most relevant to disease based on the interlinkage of gene sets and the association between gene sets and phenotypes. In this paper, WGCNA was used to cluster genes with similar expression patterns to obtain the modules most relevant to the clinical phenotype of PAAD patients.

### Construction of the multi-RNAs-based classifier

In order to select the best lncRNA and mRNA from the high-dimensional data to construct the classifier, we chose LASSO COX regression method. LASSO, a method of filtering variables, sets a penalty function to make certain coefficients zero, and some coefficients are also compressed. Thus, achieving the purpose of shrinking subsets [19]. The RNAs related to OS were identified by LASSO COX regression model. The regression coefficients (β) are used to form the multi-RNAs-based classifier = ∑ EXP(RNA) * β. After the risk scores of all patients were calculated by LASSO cox, the patients were divided into high-risk group and low-risk group with the median as the critical value. Time dependent receiver operating characteristic and the area under receiver operating characteristic curve were used to evaluate the predictive ability of the model, both of which were realized by the RMS package.

### Construction of the ceRNA network

In order to predict lncRNA downstream of miRNAs, we used the miRcode (http://www.mircode.org/index.php). MiRcode is a database that predicts the interaction between human miRNAs and various types of RNA, such as mRNA and lncRNA. StarBase (http://starbase.sysu.edu.cn/), miRDB (http://www.mirdb.org/), miRTarBase (http://mirtarbase.mbc.nctu.edu.tw/) and Targetscan (http://www.targetscan.org/) were used to locate the target mRNAs.

## Discussion

Among pancreatic tumors, PAAD has the highest incidence, accounting for about 85% of all cases, while other subtypes such as pancreatic neuroendocrine tumors account for less than 5% [20]. Because PAAD patient do not show special clinical symptoms at an early stage and cannot be detected and treated early, the 5-year survival rate is the lowest among all pancreatic tumor types [21]. It is of great importance to identify the potential molecular markers and therapeutic targets for PAAD. Therefore, in this study, we conducted a bioinformatics analysis of the data downloaded from the GCTA, ICGC and GTEx databases, and finally established a specific ceRNA network and a 14-mRNAs-based classifier for predicting the OS of PAAD patients.

A large number of studies have shown that non-coding RNAs, such as lncRNAs and miRNAs, played an important regulatory role in promoting or inhibiting the occurrence and development of cancer [22, 23]. In 2011, the ceRNA hypothesis proposed by Salmena et al. showed that lncRNA, miRNA and mRNA formed a regulatory network [12]. According to the ceRNA hypothesis, many researchers have demonstrated that lncRNA, miRNA and mRNA interact with each other as corresponding ceRNA networks during disease processes. Moreover, Wang et al. constructed a pancreatic cancer mRNA-miRNA-lncRNA sub-network by mining the GSE16515 and GSE15471 datasets, filling the gap in this area [24]. However, there were still some shortcomings in his research: First, there were only 72 PAAD and 52 normal samples included in the study. Second, only two databases were used to predict miRNA and lncRNA upstream of the mRNA. In our study, in order to make the study more credible, we used PAAD patient data from two databases, TCGA and ICGC, to establish the training cohort and the validation cohort, which respectively contained 178 and 267 samples. Moreover, data from four databases was used to predict target genes of miRNA. In addition, LASSO is a frequently used statistical method for multicollinearity problems. We used LASSO COX regression models for the screened RNAs and obtained the more refined prediction model through compression estimation to predict the OS of PAAD. In this article, we have established two different classifiers to predict the OS of patients with PAAD. Clinical RNA data from tumor tissues of PAAD patients could be combined with our established classifier to predict the prognosis of patients. Therefore, clinicians could use the predicted results as reliable reference when formulating treatment strategies for patients. The classifier based on 14 mRNAs had a high accuracy in both the training cohort and validation cohort. Furthermore, starting with these 14 mRNAs, we obtained 60 lncRNAs, 3 miRNAs and 3 mRNAs, thus constructing a specific ceRNA network. After that, we screened lncRNA in the ceRNA network and 14 mRNA of the original classifier to build a new 8-lncRNAs-8-mRNAs-based classifier. Comparing the AUC of the two classifiers, the predictive function of the two classifiers was similar, which proved that the mRNA signature alone was robust enough to discriminate patients’ prognosis without the integration of non-coding RNA. In addition, our GSEA analysis of the high-risk group showed that genes were mainly enriched in p53 signaling pathway, TGF-β signaling pathway and pancreatic tumors. In the study of Bailey P et al., the gene network of the four subtypes of pancreatic tumors all involved in TGF-β signaling pathway, among which the squamous subtype with significant P53 gene mutation has the worst prognosis [25]. This result was consistent with the low overall survival rate of the high-risk group, which also verified the accuracy of the 14-mRNAs-based classifier in predicting the OS of PAAD patients.

In recent years, some genes included in our ceRNA regulatory network have been reported in literature to be associated with regulating the development of tumors. ADAM9 is highly expressed in pancreatic ductal adenocarcinoma and is closely related to vascular invasion of cancer cells [26]. Zhu et al. reported that EFNB2 promoted the differentiation, migration and invasion of cancer cells in pancreatic ductal adenocarcinoma [27]. FEN1, a direct target of miR-140-5p, is related to the epithelial-mesenchymal transition of hepatocellular carcinoma cells [28]. MiR-125a-5p has been reported to inhibit the expression of GAB2 and Bax proteins, and ultimately inhibited the proliferation and invasion of breast cancer cells [29]. MiR-20b-5p has been extensively studied in many cancers, such as lung cancer and colorectal cancer [30, 31]. However, the role and mechanism of RNAs such as DTNA, miR-125-5p, miR-20b-5p in the occurrence and development of PAAD still need further investigation.

Furthermore, GO-GESA for green module obtained by WGCNA analysis was performed to identified metabolic pathways enriched by the genes within the module. The result showed that those mRNAs were enriched in endoplasmic reticulum (ER) stress. Study had shown that both ER stress and fold-protein response are up-regulated in pancreatic neuroendocrine tumors, which can be used as therapeutic targets for pancreatic tumors [32, 33]. Moreover, these mRNAs were also enriched in the response to the nutrient levels pathway and peptide metabolic process pathway. Abnormalities in the processes of energy metabolism, protein metabolism and fat metabolism of tumor tissues had been the focus of research in recent years [34, 35]. Analysis of the KEGG pathway revealed that these genes had significantly enrichment in the pancreatic secretion pathway. The above results indicated that these specific enriched pathways are closely related to the pathological mechanism of PAAD and could be further studied.

There were still some limitations to our study. First, although we used independent samples to validate the 14-mRNAs classifier, we did not validate the 8-lncRNAs-8mRNAs-classifier due to the lack of sufficient lncRNA data by ICGC. Second, the mechanisms of those lncRNAs, miRNAs and mRANs, included in the ceRNA interaction network, were still unclear and required further research. Third, prospective studies of larger samples in different regions need to be used to verify the accuracy of the models.

## Conclusions

We developed a lncRNA-miRNA-mRNA co-expression network from a multi-omics perspective. In addition, a multi-mRNAs-based classifier with high accuracy was built to predict the OS of PAAD patients.

## Supplementary information

**Additional file 1: Figure S1.** Gene enrichment in the high-risk group of the training cohort.

**Additional file 2: Figure S2.** 8-lncRNAs-8-mRNAs-based classifier. (A) LASSO coefficient profiles of 60 lncRNAs and 14mRNAs. A vertical line is drawn at the value chosen by 13-fold cross-validation. (B) Ten-time cross-validation for tuning parameter selection in the LASSO model. (C) Kaplan–Meier survival analysis of the 8-lncRNAs-8-mRNAs-based classifier was performed. (D) Time-dependent ROC curve of the 8-lncRNAs-8-mRNAs-based classifier was performed.

## Data Availability

The datasets used during the current study are available from the corresponding author on reasonable request.

## Abbreviations

PAAD: Pancreatic adenocarcinoma
ceRNA: Competing endogenous RNA
miRNAs: MicroRNA
MREs: MicroRNA response elements
lncRNAs: Long noncoding RNAs
TCGA: The Cancer Genome Atlas
GTEx: Genotype-tissue expression
WGCNA: Weighted correlation network analysis
TMM: Trimmed mean of M values
GO: Gene ontology
BP: Biological processes
CC: Cellular component
MF: Molecular function
KEGG: Kyoto Encyclopedia of Genes and Genomes
GSEA: Gene Set Enrichment Analysis
TOM: Topological overlap matrix
tdROC: Time dependent receiver operating characteristic
AUC: Area under receiver operating characteristic curve
