# Correction to: Identification of a competing endogenous RNA network associated with prognosis of pancreatic adenocarcinoma

**DOI:** 10.1186/s12935-020-01381-x

**Published:** 2020-07-01

**Authors:** Wanqing Weng, Zhongjing Zhang, Weiguo Huang, Xiangxiang Xu, Boda Wu, Tingbo Ye, Yunfeng Shan, Keqing Shi, Zhuo Lin

**Affiliations:** 1 Zhejiang Provincial Key Laboratory, The First Affiliated Hospital of Wenzhou Medical University, Wenzhou, 325000 Zhejiang People’s Republic of China; 2 Precision Medicine Center Laboratory, The First Affiliated Hospital of Wenzhou Medical University, Wenzhou, 325000 Zhejiang People’s Republic of China; 3 Department of Hepatobiliary Surgery, The First Affiliated Hospital of Wenzhou Medical University, Wenzhou, 325000 Zhejiang People’s Republic of China; 4 Department of Liver Diseases, The First Affiliated Hospital of Wenzhou Medical University, Wenzhou, 325000 Zhejiang People’s Republic of China

## Correction to: Cancer Cell Int (2020) 20:231 10.1186/s12935-020-01243-6

Following publication of the original article [1], the authors notified us that Fig. 6 was incorrectly submitted. The correct figure is presented below.

**Fig. 6:**
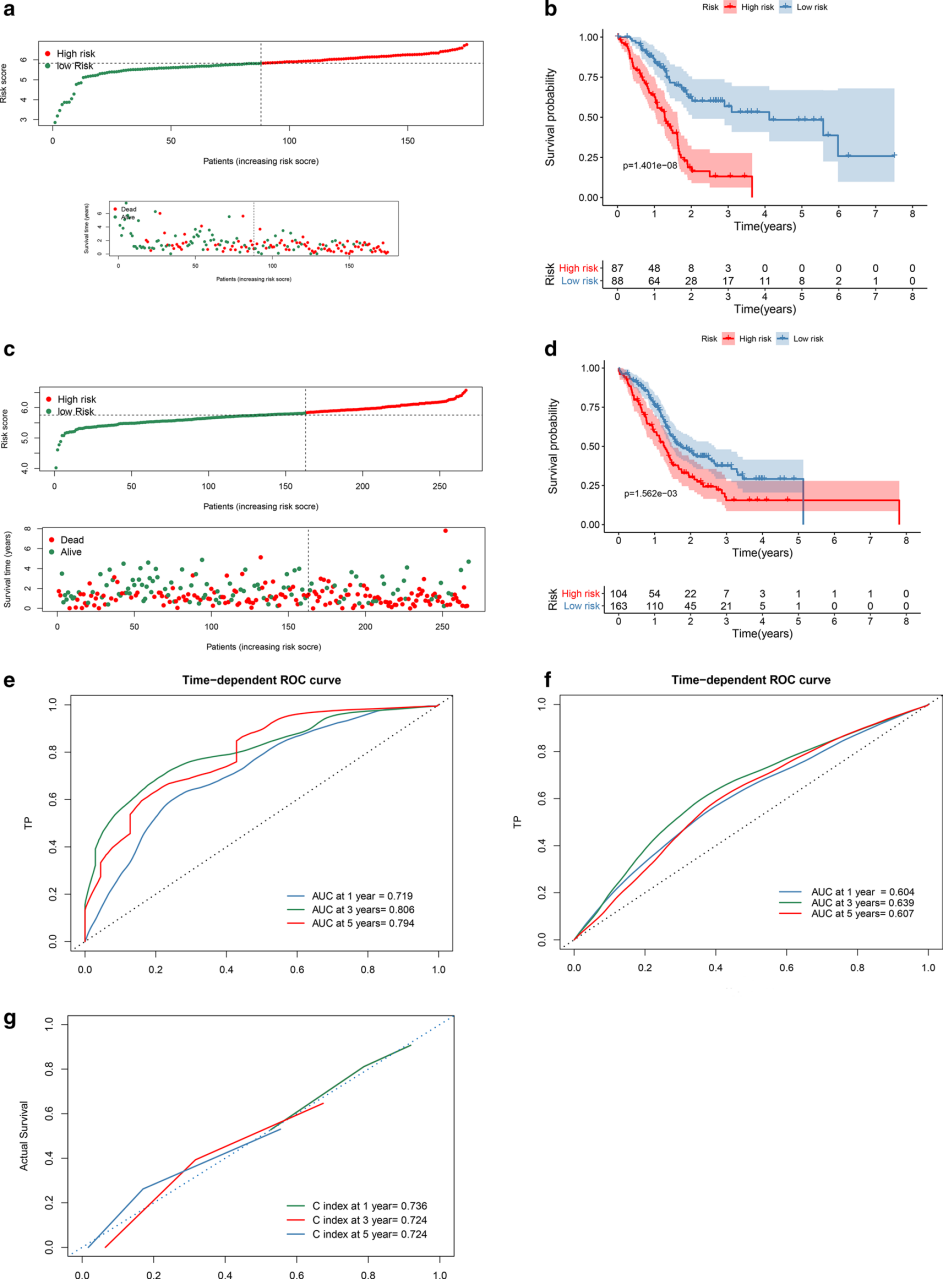
Multi-mRNAs-based classifier. **a**, **b** PAAD patients in the training cohort and the validation cohort were classified into predicted low and high-risk groups according to the multi-mRNAs-based classifier. **b**, **d** Kaplan–Meier survival analysis of the multi-mRNAs-based classifier was performed. **e**, **g** Time-dependent ROC curve and calibration curves of the multi-mRNAs-based classifier in the training cohort. **f** Time-dependent ROC curve of the multi-mRNAs-based classifier in the validation cohort
