# Supplementary figures and images for: Identification of a competing endogenous RNA network associated with prognosis of pancreatic adenocarcinoma
Source: Cancer Cell Int. 2020 Jun 11;20:231. doi: 10.1186/s12935-020-01243-6 (PMC7288603; doi:10.1186/s12935-020-01243-6)

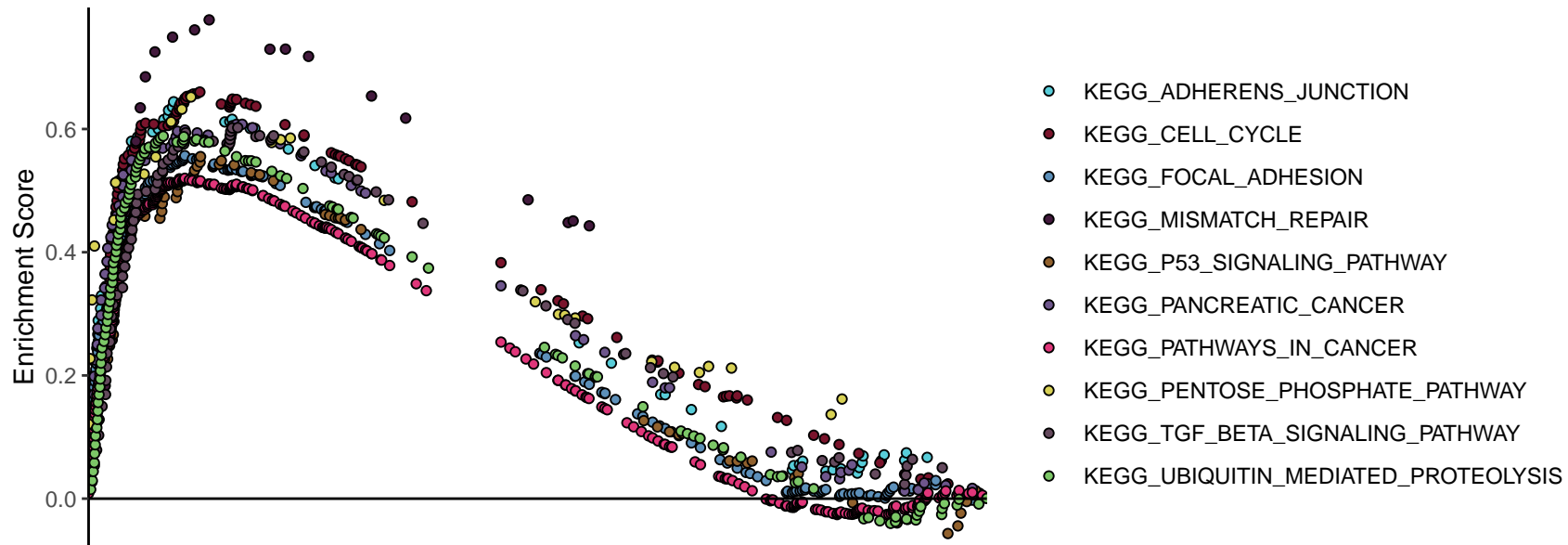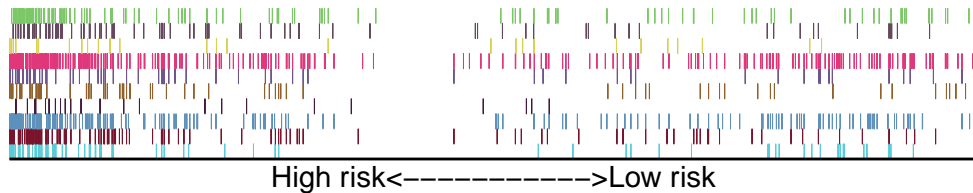

Supplement: Supplementary file 1 — Additional file 1: Figure S1. Gene enrichment in the high-risk group of the training cohort. [file 12935_2020_1243_MOESM1_ESM.pdf]

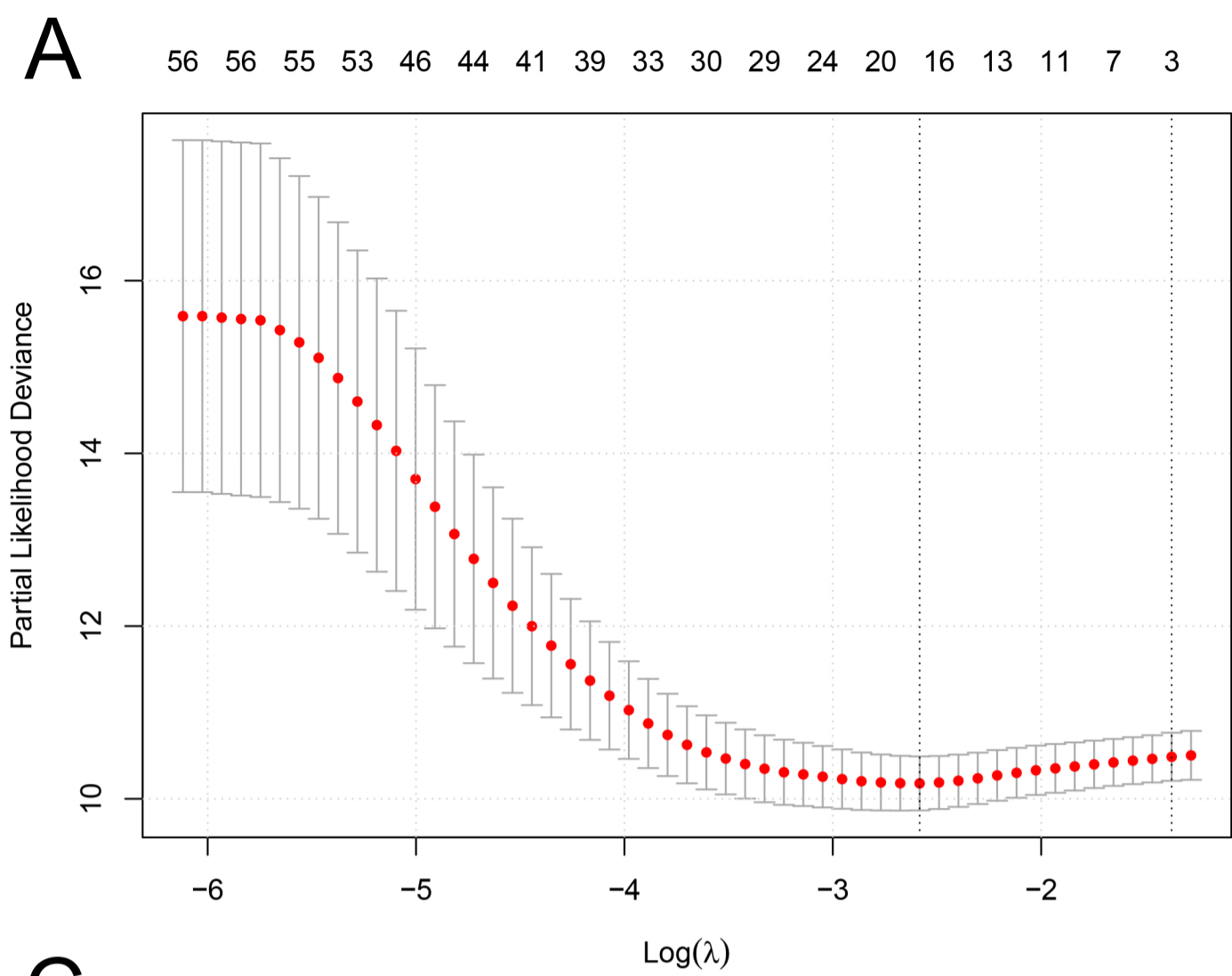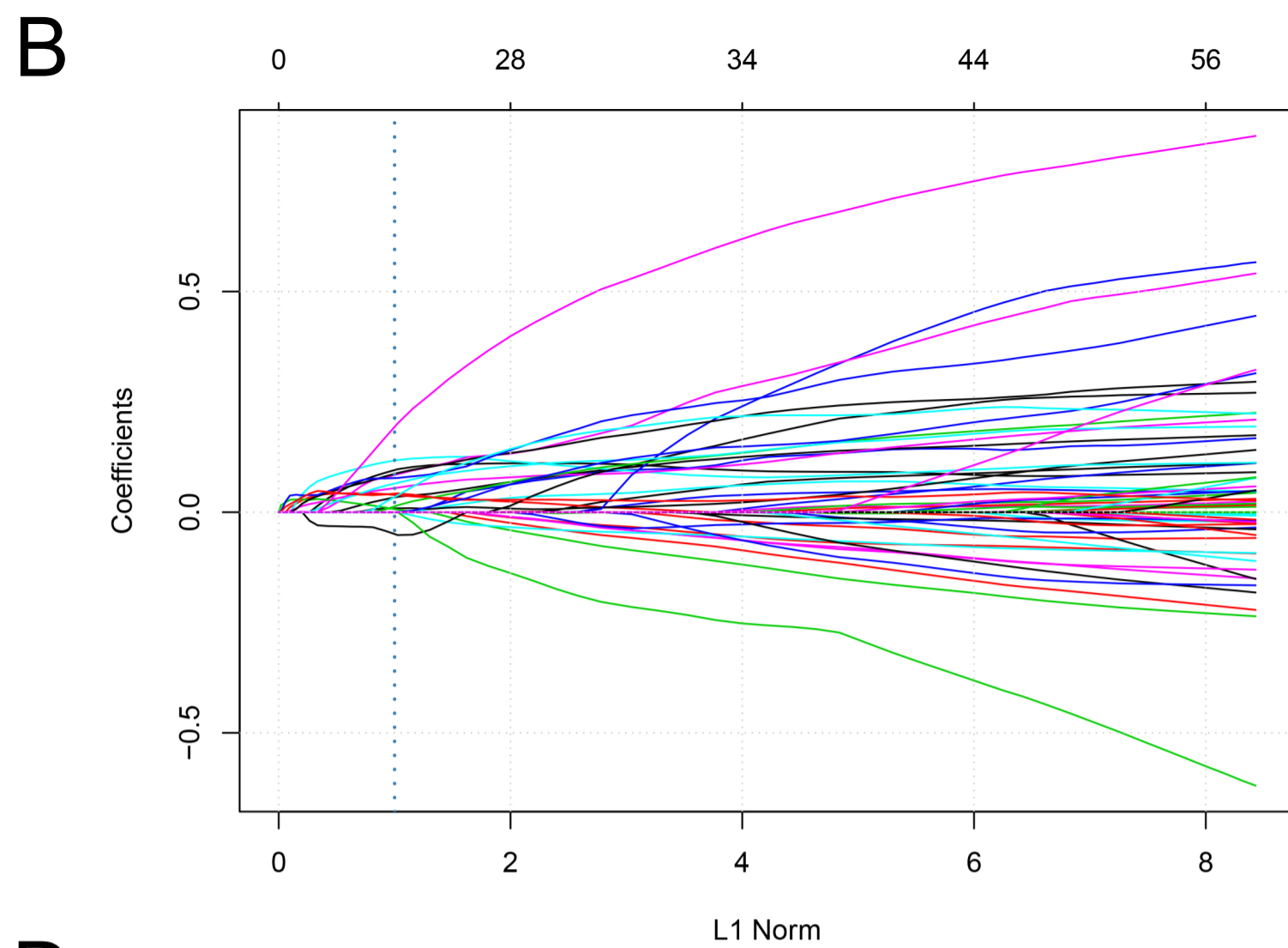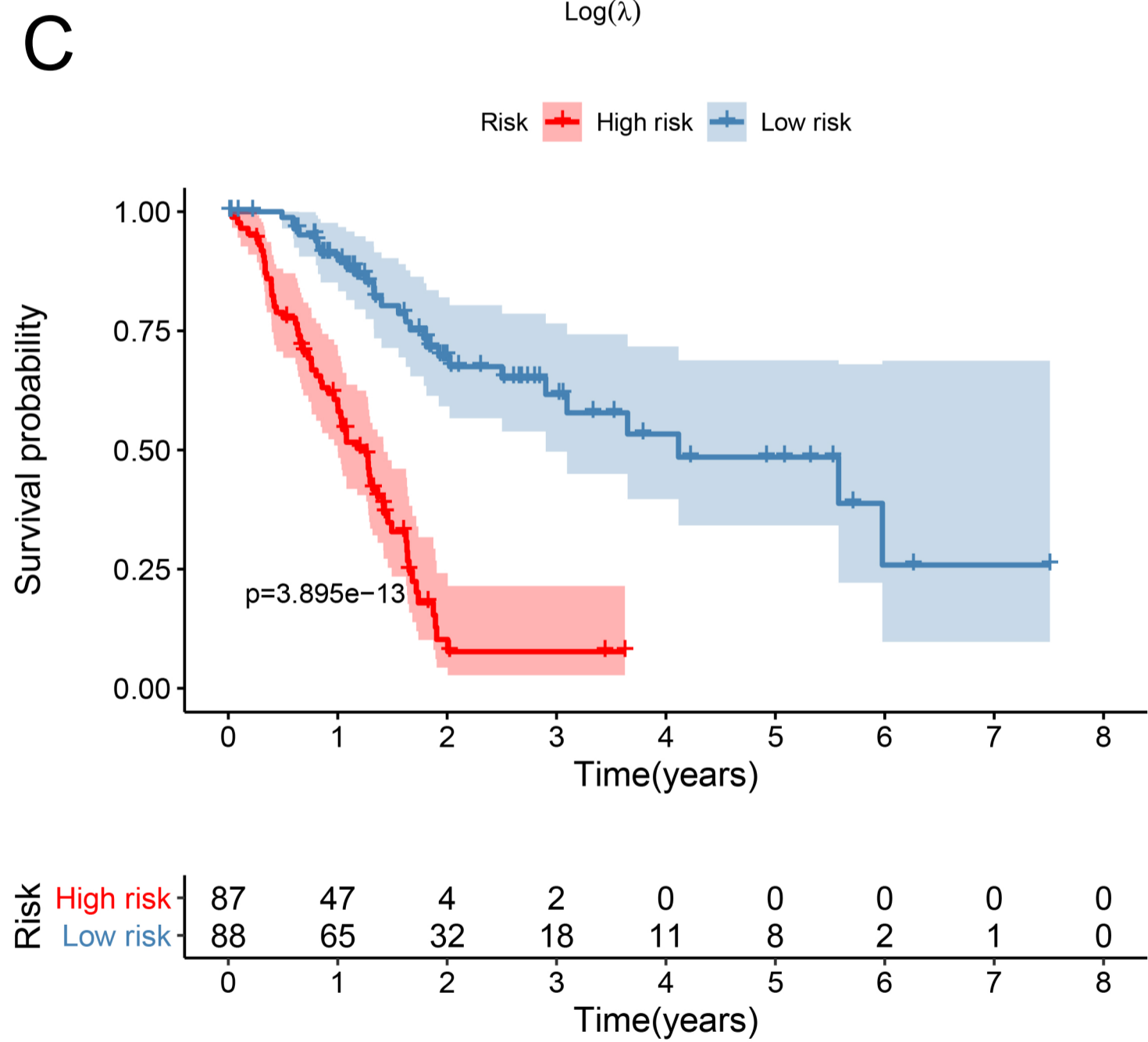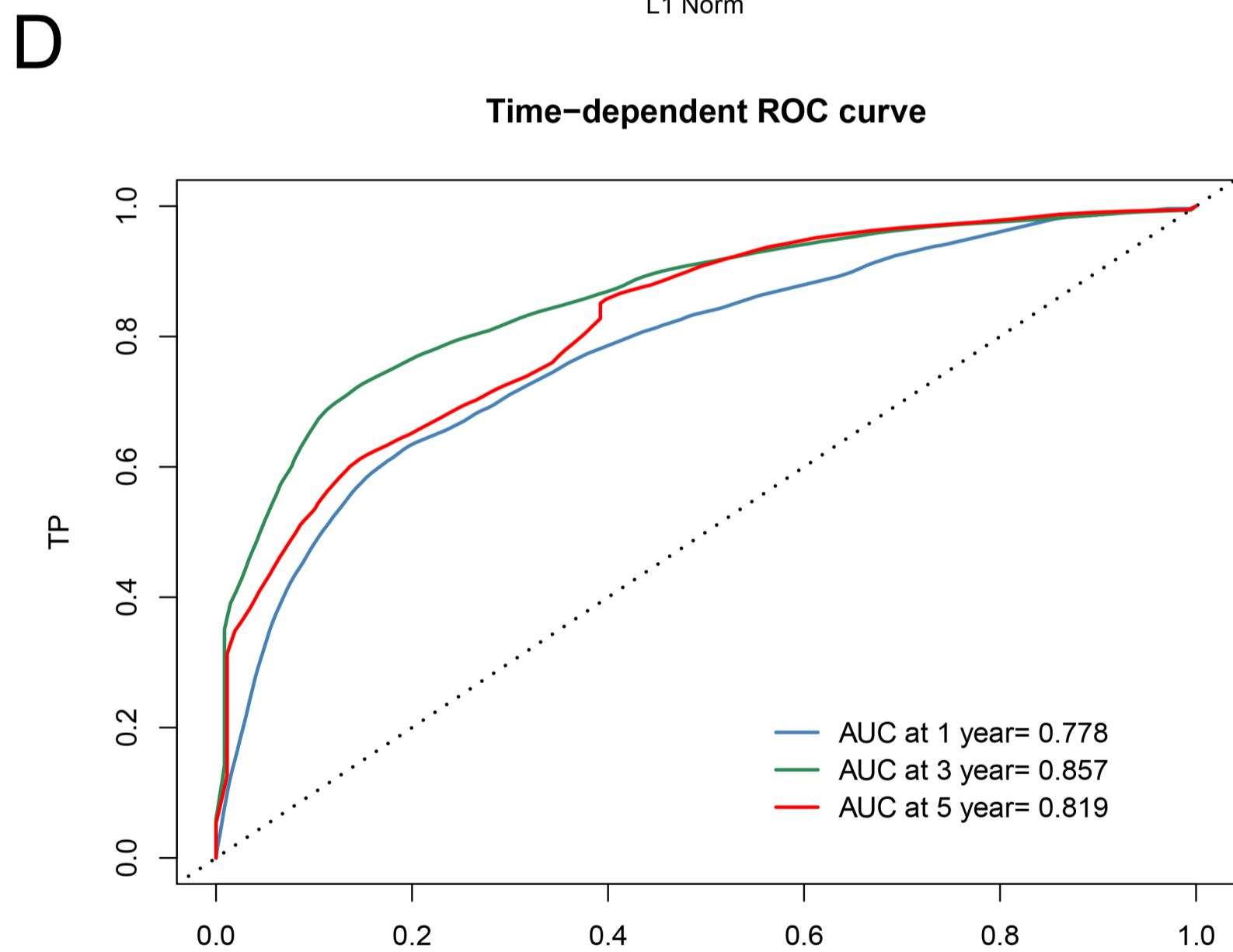

Supplement: Supplementary file 2 — Additional file 2: Figure S2. 8-lncRNAs-8-mRNAs-based classifier. (A) LASSO coefficient profiles of 60 lncRNAs and 14mRNAs. A vertical line is drawn at the value chosen by 13-fold cross-validation. (B) Ten-time cross-validation for tuning parameter selection in the LASSO model. (C) Kaplan–Meier survival analysis of the 8-lncRNAs-8-mRNAs-based classifier was performed. (D) Time-dependent ROC curve of the 8-lncRNAs-8-mRNAs-based classifier was performed. [file 12935_2020_1243_MOESM2_ESM.pdf]
